# Antibiotic Resistance of Profiles of *Escherichia coli* Strains From Imported Chicks and Eggs in the Semi‐Intensive Poultry Sector of Kinshasa, Democratic Republic of the Congo

**DOI:** 10.1155/vmi/1141830

**Published:** 2026-07-28

**Authors:** Branham Kitoko, Soledad Colombe, Tatiana Banze, Patrick Ngoie, Papy Katembo, Prince Kimpanga, Joseph Mabi, Justin Masumu

**Affiliations:** ^1^ Faculty of Veterinary Medicine, National Pedagogical University, Kinshasa, Democratic Republic of the Congo; ^2^ Interdisciplinary Center for Health Risk Management, Kinshasa, Democratic Republic of the Congo; ^3^ Central Veterinary Laboratory of Kinshasa, Kinshasa, Democratic Republic of the Congo; ^4^ Unit of Emerging Infectious Diseases, Department of Public Health, Institute of Tropical Medicine, Antwerp, Belgium, itg.be; ^5^ Faculty of Veterinary Medicine, Catholic University of Graben (UCG), Butembo, Democratic Republic of the Congo; ^6^ Kinshasa School of Public Health, University of Kinshasa, Kinshasa, Democratic Republic of the Congo, unikin.ac.cd; ^7^ National Institute for Biomedical Research, Kinshasa, Democratic Republic of the Congo

**Keywords:** antibiotics, chicks, eggs, *Escherichia coli*, Kinshasa

## Abstract

Poultry production in the Democratic Republic of Congo can be divided into three sectors: large‐scale, semi‐intensive, and backyard production. Kinshasa, capital city of the Democratic Republic of Congo, hosts a number of large‐scale and semi‐intensive farms that import the majority of their day‐old chicks and fertilized eggs. These imports are carried out legally, and both the chicks and eggs arrive by air. They could introduce pathogenic bacteria, including antibiotic‐resistant strains. This study aimed to assess the level of antibiotic resistance in *E. coli* strains isolated from fecal samples of imported chicks and locally hatched chicks from imported eggs in the semi‐intensive poultry production system in Kinshasa. This study was conducted from September 15, 2019, to April 15, 2020. Five (56%) importers and hatcheries based in Kinshasa agreed to participate and to inform the team of incoming batches. The team was made aware of five batches. Cloacal samples were collected from egg‐laying chicks imported from Belgium (two batches), broiler chicks imported from Zambia (two batches), and broiler chicks hatched locally from incubated eggs imported from an unidentified country (one batch). Samples underwent biochemical identification for *E. coli* and antimicrobial resistance testing using the disk diffusion method. *E. coli* was detected in all cloacal samples. Antibiotic susceptibility testing revealed high resistance to amoxicillin (76%), trimethoprim/sulfamethoxazole (75%), and ampicillin (69%), but little to no resistance to cefuroxime (0%), gentamicin (20%), and cefixime (24%). All imported batches were resistant to at least one antibiotic, with the strains from Belgium and Zambia exhibiting two and five different resistance profiles, respectively. This study demonstrates that the importation of chicks and fertilized eggs into the DRC can lead to the introduction of multidrug‐resistant *E. coli* strains.

## 1. Introduction

*Escherichia coli* is a bacterium that belongs to the genus *Escherichia* in the *Enterobacteriaceae* family and Enterobacterales order. Most strains of this species are commensals of the intestinal flora of warm‐blooded animals and humans. However, some strains of *E. coli* can be pathogenic, leading to gastroenteritis and urogenital infections in humans [1].

In poultry, avian pathogenic *E. coli* (APEC) infection results in avian colibacillosis, which, although considered a secondary infection, causes major economic losses in poultry farms [2].

Antibiotic therapy is often recommended to prevent avian colibacillosis [3], and the uncontrolled use of antibiotics is a major factor contributing to the emergence and spread of acquired resistance traits in both pathogenic and nonpathogenic bacteria [1, 4]. In food‐producing animals, such as chickens, this resistance selection pressure is exacerbated by the use of antibiotics for prevention, treatment, and growth promotion for the entire flock [5].

The development of antibiotic resistance, particularly among *E. coli* in poultry farming, is a major global concern with a substantial impact on public health [1, 6].

*E. coli* strains are considered important indicators of antibiotic resistance. Since they are part of the normal microbiota of warm‐blooded animals and humans [7], the emergence of resistance in a nonpathogenic strain can act as a reservoir and horizontally transmit resistance genes to other pathogenic bacteria, both within and across animal species, including humans [6, 8].

Locally herding poultry in Kinshasa is expensive. Eighty percent of animal feed is still imported, and farmers strongly depend on the availability of medical products for both growth enhancement and veterinary health. This contributes to the high market price of locally raised chickens compared to imported frozen products. As a result, the import of frozen slaughtered chickens still dominates the chicken food market. In order to support the local economy, local production of broilers and layers has been highly promoted [9]. The poultry sector in the DRC can be categorized into three main channels: large‐scale, semi‐intensive, and backyard production. The poultry sector includes production of meat, eggs, and live animals (broilers and layers). This article focuses on live animals.

Nearly 80% of chicken farming is traditional, through backyard production [10, 11] and most of these flocks can be found in rural and periurban areas. The chickens are primarily consumed in their production environments, although a small proportion is transported to larger consumption markets. Backyard poultry production remains largely unstructured, relying on natural reproduction with limited attention to animal health and nutrition. Small‐ and medium‐scale farms are also affected by high input costs, which limit their capacity to sustain operations and scale up production [12].

In Kinshasa, the capital of the DRC (approximately 15 to 17 million inhabitants), as well as in other large Congolese cities such as Lubumbashi or Goma, the breeding of improved, fast‐growing breeds is practiced on large and semi‐intensive scales. There are 10 large‐scale poultry farms (> 50,000 birds overall) mainly located around Kinshasa, Lubumbashi, and Goma. In Kinshasa, the four large‐scale farms are « Minoterie du Congo (Mino Congo)/flour mill of Congo», «Minoterie de Matadi (MIDEMA)/flour mill of Matadi», «Association de Lutte contre la Pauvreté Fortune de Dieu (ALPFD)/Association for the Fight against Poverty “Fortune of God”», and the Domaine Industriel présidentiel de la N’sele (DAIPN)/N’sele Presidential Industrial Domain ‘». The number of semi‐intensive poultry farms varies over time.

Large‐scale and semi‐intensive poultry farming is highly dependent on the importation of day‐old chicks and fertilized eggs, partly due to the limited number of local breeding centers and hatcheries [12]. Although comprehensive official data are lacking, it is estimated that fewer than ten hatcheries operate nationwide, with insufficient capacity to meet the national demand. In Kinshasa, the organization of health monitoring in poultry farms varies according to flock size [13]. In small holdings (fewer than 100 birds), farmers themselves manage animal health. Antibiotics are frequently administered without veterinary consultation, with deviations from recommended doses and treatment durations or schedules. Moreover, some antibiotics are also used as growth promoters, a poorly regulated practice that may contribute to the selection of antibiotic‐resistant bacteria [5].

A recent study conducted in poultry farms in Kinshasa revealed a predominance of *E. coli* isolates (94%) with high resistance rates to amoxicillin (83.3%), ampicillin (83.3%), and sulfadimidine (83.3%). Conversely, all analyzed strains were sensitive to gentamicin and cefuroxime (100%) [14].

Given the risk of transovarial transmission of *E. coli* through penetration of the eggshell [15], it is critical to investigate whether the recurrent importation of chicks and eggs in the DRC poses a risk of introduction of antibiotic‐resistant *E. coli* strains. We aimed to assess the prevalence and level of antibiotic resistance in *E. coli* strains isolated from cloacal samples from imported chicks and locally hatched chicks after incubating imported eggs within the semi‐intensive production. Understanding the risk of introduction of antibiotic resistance is essential for evaluating implications for poultry health and public safety.

## 2. Methods

### 2.1. Study Site

**FIGURE 1:**
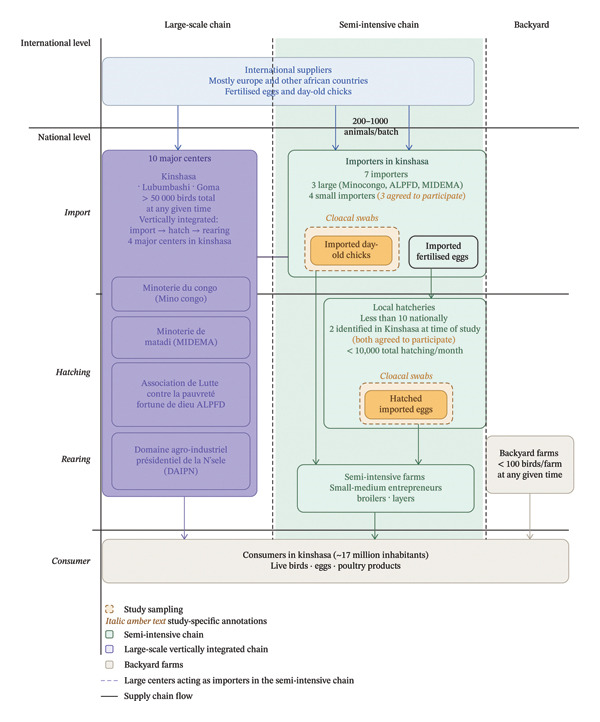
Graphic summary of live poultry production in Kinshasa, DRC, 2020.

This study took place in Kinshasa, capital city of the DRC, from September 15, 2019, to April 15, 2020. It was conducted within the semi‐intensive poultry sector, specifically among firms importing chicks and local producers of chicks from imported eggs (named hatcheries throughout the rest of the document). Kinshasa’s 2020 population was estimated at 17,032,322 (World Population Review, 2024). Approximately 10 hatcheries of semi‐intensive scale production currently exist in the DRC, notably in Kinshasa, Lubumbashi, and Goma. These facilities handle less than 10,000 eggs for hatching per month. The importation of eggs for hatching, as well as day‐old chicks from foreign countries is carried out by specialized importers. Most day‐old chicks and eggs are imported by three large companies, namely, «Minoterie du Congo (Mino Congo)/flour mill of Congo», “Minoterie de Matadi (MIDEMA)/flour mill of Matadi», and «Association de Lutte contre la Pauvreté Fortune de Dieu (ALPFD)/Association for the Fight against Poverty “Fortune of God”». There are also a few smaller importers (4 at the time of the study) importing in total 1000 to 5000 eggs or chicks/month [16, 17]. These importing companies then subsequently sell the imported products to hatcheries and farms. The live‐poultry production in the DRC is graphically summarized in Figure 1. At the time of the study, there were 7 importers in Kinshasa (Mino Congo, ALPFD, MIDEMA, as well as 4 small importers). Two semi‐intensive hatcheries could be identified in Kinshasa.

### 2.2. Study Design

The study population consisted of imported chicks and locally hatched chicks after incubation of imported eggs within the semi‐intensive sector (before being handed over to buyers, see Figure 1).

We conducted a descriptive cross‐sectional study. A nonprobability sampling method based on the “snowball” sampling technique was used. Initially, the addresses of potential chick importers were communicated to us either by the hatcheries themselves, by government departments in charge of livestock and fisheries, or by importers consulted earlier. The same strategy was applied to importers of fertilized eggs. In total, seven importers (Mino Congo, ALPFD, MIDEMA, and 4 small importers) and two hatcheries were identified in Kinshasa and invited to participate in the study. Three small importers (1000 to 5000 chicks/eggs) and the two hatcheries ultimately agreed to participate.

It was agreed that the importer/hatchery would inform the study team whenever they were about to receive a new shipment. The study team also regularly called the importers/hatcheries to remind them of the study and to follow‐up on shipment dates. The size of the import batches (=group of chicks or eggs received in one import) varied depending on demand and import conditions at that time, generally ranging between 200 and 1000 chicks/eggs per batch. The exact number of chicks/eggs within a batch could not be determined, as part of the batch was sometimes sold on the way from the airport to the storage unit where batches are stored before being dispatched to the main hatcheries.

The chicks included in the study were those imported on the day, directly brought from the airport to the storage unit and therefore not yet installed in the breeding buildings to avoid possible contamination through the local environment. Similarly, chicks hatched from imported eggs were included in the study at the time of hatching or before transfer to the breeding building.

All imported chicks or chicks incubated from imported eggs that were found already transferred to the rearing house were excluded from the study.

### 2.3. Data Collection

For each batch of chicks/hatched eggs, we selected 20 animals (out of 200–1000) at random and collected individual fecal samples using a cloacal swab. Individual cloacal sampling was preferred as it allows precise attribution of bacterial isolates and antimicrobial resistance profiles to specific animals, while avoiding the limitations associated with pooled samples from transport boxes, particularly the risk of cross‐contamination between individuals. Samples were transported directly in a cooler box to the laboratory within 2 h for analysis. For all batches sampled, information on the origin of the chicks, breed, and age (in days) was collected.

### 2.4. Ethical Approval

Ethical approval has been granted by the scientific committee of the Faculty of Veterinary Medicine of the National Pedagogical University (UPN)/DR Congo (approval no. FMV/SC/053/2019).

### 2.5. Analysis

We performed all bacteriological analyses at the Central Veterinary Laboratory in Kinshasa.

### 2.6. Isolation and Identification of *E. coli*

Fecal swabs were pre‐enriched in buffered peptone water (BPW) at 37°C overnight. *E. coli* isolation was performed using standard bacteriological methods; the culture suspension was grown on Mac Conkey agar at 37°C for 24 h. Presumed pink *E. coli* colonies were cultured on eosin‐methylene blue (EMB) agar after incubation for 24 h at 37°C. Colonies showing a metallic green or fluorescent reflection on EMB underwent biochemical identification tests by conventional galleries (Urea‐Indole, Mannitol‐Mobility, Kligler, and Simmons Citrate). Isolates showing indole‐positive, methyl‐red‐positive, and citrate‐negative characteristics were retained as *E. coli* [18, 19].

### 2.7. Antibiotic Susceptibility Testing

Antibiotic susceptibility was assessed using the disk diffusion method in accordance with the 2021 recommendations of the Antibiogram Committee of the French Society of Microbiology [20]. The turbidity of a suspension prepared from a 24‐h culture colony was adjusted to a 0.5 Mac Farland standard using sterile normal saline. Antimicrobial susceptibility testing was conducted on Mueller–Hinton agar incubated at 37°C for 24 h. Each of the 100 isolates was tested against amoxicillin, trimethoprim/sulfamethoxazole, ampicilin, cefimixine, gentamicin, and cefuroxime.

The diameters of the zones of inhibition were measured with a ruler, following the 2021 recommendations of the Antibiogram Committee of the French Society of Microbiology [20]. Antibiogram results were interpreted in two clinical categories, sensitive and resistant. All isolates in the high exposure sensitive (HES) clinical category were classified as sensitive.

### 2.8. Data Analysis

We calculated frequencies and proportions of chicks resistant and susceptible to different antibiotics according to the country of origin. The level of resistance of isolated *E. coli* strains to each antibiotic was estimated as the proportion of strains isolated from the samples that grew in the presence of a specific antibiotic.

## 3. Results

### 3.1. Description of Samples

Throughout the study period, we were informed from hatchers and importers of five incoming and confirmed shipments. We collected samples from all five shipments, four from imported chicks and one from imported eggs. For each of the five batches, 20 fecal samples were collected, making a total of 100 samples over the five batches, i.e., 80 samples from imported chicks and 20 samples from chicks hatched locally from imported eggs.

The imported chicks were imported from Belgium (two batches) and Zambia (two batches), while the country of origin of the batch of eggs hatched locally in Kinshasa remained unknown. The breed distribution showed that 60% of the imported chicks/eggs were of the Cobb500 breed (broiler chicks) and 40% of the Lomman brown breed (egg‐laying chicks) (Table 1).

**TABLE 1:** Summary of samples according to the country of origin, breed, and purpose, imported chicks, and fertilized eggs, Kinshasa, 2020.

Batch number	Number of chicks sampled	Country of origin	Breed	Purpose
1	20	Unknown^∗^	Cobb 5000	Broiler chicks
2	20	Zambia	Cobb 500	Broiler chicks
3	20	Zambia	Cobb 500	Broiler chicks
4	20	Belgium	Lomman Brown	Egg‐laying chicks
5	20	Belgium	Lomman Brown	Egg‐laying chicks

^∗^These chicks came from eggs imported from outside the DRC and then incubated in the DRC. The country of import was not revealed.

### 3.2. Bacterial Identification and Antibiotic Susceptibility Testing of *E. coli* Isolates

**FIGURE 2:**
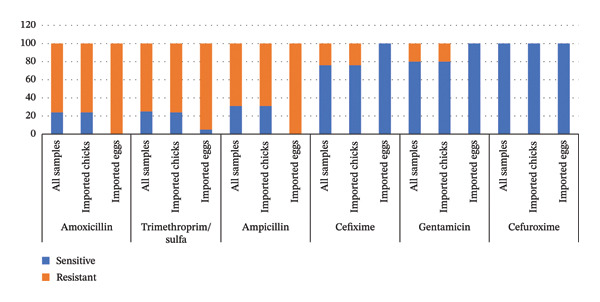
Proportion (%) of antibiotic resistance in *E. coli* strains isolated from imported chicks and hatched imported eggs, Kinshasa, DRC, 2020.

After isolation and identification, *E. coli* colonies grew in all 100 cultured samples (100%). As shown in Figure 2, the highest resistance was observed with amoxicillin (76% of the 100 isolated strains), followed by trimethoprim/sulfamethoxazole (75%) and ampicillin (69%), while 100% sensitivity was observed with cefuroxime, followed by gentamicin (80%) and cefixime (76%).

### 3.3. Antibiotic Resistance Profiles of *E. coli* Strains

We characterized 4 different antibiotic resistance profiles (A–D in Table 2). Chicks hatched in the DRC from imported fertilized eggs all presented the same pattern of resistance to three of the six antibiotics tested (amoxicillin, trimethoprim/sulfamethoxazole, and ampicillin, Profile A).

**TABLE 2:** Antibiotic resistance profiles by chick breed, imported chicks, and fertilized eggs, Kinshasa, DRC, 2020.

Batch number	Resistance profile	Country of origin	Breed	Purpose	A1^∗^	A2^∗^	G^∗^	C1^∗^	TS^∗^	C2^∗^	Number of antibiotics the strain was resistant to (proportion)
1	A	Unknown	Cobb500	Broiler	1	1	0	0	1	0	3 (50%)
2	B	Belgium	L. brown	Egg‐laying	1	1	1	0	0	1	4 (67%)
3	A	Belgium	L. brown	Egg‐laying	1	1	0	0	1	0	3 (50%)
4	A	Zambia	Cobb500	Broiler	1	1	0	0	1	0	3 (50%)
5	A	Zambia	Cobb500	Broiler	1	1	0	0	1	0	3 (50%)
5	C	Zambia	Cobb500	Broiler	0	0	0	0	1	0	1 (17%)
5	A	Zambia	Cobb500	Broiler	1	1	0	0	0	1	2 (50%)
5	D	Zambia	Cobb500	Broiler	0	0	0	0	0	1	1 (17%)
Proportion of batches that presented a resistance to the respective antibiotic					4/5	5/5	1/5	0/5	4/5	2/5	

*Note:* ^∗^A1: ampicillin; A2: amoxicillin; G: gentamicin; C1: cefuroxime; TS: trimethoprim/sulfamethoxazole; C2: cefixime.

Abbreviation: L. Brown, Lomman Brown.

The two batches of chicks from Belgium presented different resistance profiles of 67% and 50% each: The first batch presented resistance to ampicillin, amoxicillin, gentamycin, and cefixime (Profile B), while the second batch presented the same resistance profile as the batch of chicks from eggs imported and incubated in the DRC, i.e., to amoxicillin, trimethoprim/sulfamethoxazole, and ampicillin (Profile A).

The two batches of chicks from Zambia presented three different antibiotic resistance profiles. The chicks from the first batch presented a single resistance profile, while those from the second batch exhibited three. The first batch was resistant to three antibiotics (ampicillin, amoxicillin, and trimethoprim/sulfamethoxazole) out of the six tested (Profile A). Of the four profiles from the second batch, two also presented with Profile A. The other two profiles were resistant to one of the six antibiotics tested (17%): Trimethoprim/sulfamethoxazole (Profile C) and Cefixime (Profile D). These results are presented in Table 2.

## 4. Discussion

To our knowledge, this is the first study conducted in the DRC analyzing antibiotic resistance profiles of strains of *E. coli* from imported chicks and eggs. In the DRC, most day‐old chicks are imported from the Netherlands, Belgium, Zambia, and Congo‐Brazzaville [16]. Most imports arrive and are consumed in Kinshasa, though Lubumbashi and Goma increasingly also farm imported chicken breeds and chickens produced locally after incubating imported eggs.

We showed that imported day‐old chicks and eggs in the DRC can constitute a potential source of dissemination of antibiotic‐resistant *E. coli*. This global spread of antibiotic‐resistant bacteria represents a potential threat to public health. Chicks contaminated with antibiotic‐resistant *E. coli* could contaminate local livestock and lead to recurrent and persistent antibiotic resistance in the country. This resistance can be transmitted to humans through the consumption of food animals, such as poultry, or through direct contact with contaminated poultry or their by‐products [21, 22].

In our study, *E. coli* was isolated from all the cloacal samples analyzed. The isolation of *E. coli* from chicks, broilers, and laying hens produces divergent results depending on the studies but is usually expectedly quite high since *E. coli* is part of the commensals of the digestive tract of healthy animals representing about 80% of the intestinal flora. The isolation of *E. coli* was expected. In Tanzania, all broilers and laying hens in the Arusha and Mwanza regions tested in Kiiti et al.’s [5] study were positive for *E. coli*. In Lusaka, Zambia, and Sierra Leone, *E. coli* isolation rates were reported to be higher than 90% in apparently healthy laying hens [23, 24]. Lower isolation rates were detected in Benin from Belgian chicks (50%) [22], and in Egypt, from Turkish flocks (25.6%), and from Dutch chicks (44%) [21]. Differences in the prevalence of colonization can be explained in the different studies in part by differences in age and thus in the diversity of the commensal flora. Contamination of the poultry can occur both by vertical or horizontal transmission [22, 25].

The relevance of our study finding lies not in the presence of *E. coli* but in the observed antimicrobial resistance patterns. *E. coli* is an indicator microorganism for assessing the profile of antibiotic resistance within a bacterial population because of the frequency of its detection by conventional isolation and identification techniques [23]. Our study revealed high levels of antibiotic resistance of up to 76% against amoxicillin, trimethoprim/sulfamethoxazole, and ampicillin. These results are lower than those in Zambia, Tanzania, and Ethiopia where a high level of resistance of more than 75% to trimethoprim/sulfamethoxazole was found [1, 5, 26]. The high resistance levels to trimethoprim/sulfamethoxazole observed in all studies could be explained by the use of this antibiotic as a food additive [27]. The level of resistance observed to amoxicillin, trimethoprim/sulfamethoxazole, and tmpicillin is consistent with Van Boeckel’s claim that repeated exposure to low doses of antibiotics contributes to creating ideal conditions for the emergence and spread of resistant bacteria in animals [28]. Mudenda et al. assert that the resistance of *E. coli* strains to ampicillin may indicate misuse of penicillin in the poultry sector [23]. In addition, all these molecules are often used to prevent omphalitis and enteritis, and as growth promoters in food or for prophylactic treatments in chickens. Nunan et al. attest that prophylactic antibiotic use has been observed in chicken farms in Belgium, supporting the hypothesis that poor or excessive antibiotic use is one of the factors in the emergence of resistance [29]. The use of in‐ovo antibiotics in developed countries might also contribute to the development of antibiotic‐resistant strains in animals from a young age [30].

A high diversity of strains was observed in antibiotic‐resistant *E. coli* isolates depending on the origin of the chicks. A single resistance profile was observed in chicks hatched in the DRC, whereas chicks from Belgium exhibited two resistance profiles: one resistant to ampicillin, amoxicillin, gentamicin, and cefixime and the other resistant to amoxicillin, trimethoprim/sulfamethoxazole, and ampicillin.

A high diversity of resistant strains was observed among chicks from Zambia, including a profile resistant to ampicillin, amoxicillin, and trimethoprim/sulfamethoxazole. Among the three profiles identified in the second batch, one also corresponded to Profile A. The remaining two profiles were resistant to only one of the six antibiotics tested (17%), namely, trimethoprim/sulfamethoxazole (Profile C) and cefixime (Profile D). Single resistance profiles, especially involving cefixime, have been previously documented [31]. The diversity of resistance profiles observed in batches from Zambia is likely explained by the fact that a single imported batch may contain chicks/eggs originating from different farms.

Reassuringly, high levels of sensitivity were observed against cefixime (76%), gentamicin (80%), and cefuroxime (100%), a similar result as in Zambia [23], Ethiopia, and Benin where sensitivities above 70% were observed against gentamicin, cefotaxime, and ceftriaxone (two antibiotics of the cephalosporin family) [1, 22]. The high level of sensitivity against gentamicin could be explained by the fact that this molecule is not often used due to its high cost and its difficult administration in poultry [32].

This study needs to be interpreted in light of some limitations. Convenient sampling was used, due to importation restrictions during the period of the COVID‐19 pandemic. This implies that the results of our study might not be representative of all imported eggs and chicks in the DRC. However, the detection of high levels of drug‐resistant *E. coli* remains concerning, and there is no reason that our sampling might be biased toward more colonized or more resistant animals/bacteria. In addition, we used a reduced panel of antibiotics, consequently, the resistance profiles observed reflect only the antimicrobial agents tested and may not capture the full diversity of resistance phenotypes that could be detected using a broader antibiotic panel. Further studies should investigate resistance to other antibiotics. This study revealed high levels of antibiotic resistance in *E. coli* strains from imported poultry. The importance of bacterial resistance observed in imported chicks and fertilized eggs in this study indicates that the use of antibiotics in production animals contributes to the enrichment and propagation of resistance genes, highlighting the need to control the spread of resistance throughout the production chain. The study initially assumed that *E. coli*, as part of commensals of the animal digestive tract, would not present resistance to antibiotics, particularly in young healthy subjects. The level of resistance observed in this study constitutes a significant public health concern due to the potential risk of transmission of antibiotic‐resistant strains from chickens and eggs to humans and the spread of resistance to local livestock. Further studies investigating resistance to a broader range of antibiotics and analyzing mechanisms of resistance are recommended.

## Author Contributions

Branham Kitoko, Papy Katembo, and Justin Masumu wrote the study protocol; Branham Kitoko, collected the samples, Branham Kitoko, and Tatiana Banze analyzed the samples; Patrick Ngoie performed the statistical analyses and interpretation; Branham Kitoko, Prince Kimpanga, Justin Masumu, and Joseph Mabi led the drafting of the article in French; Soledad Colombe ensured translation and finalization in English.

## Funding

This study was supported by the Directorate General for Development Cooperation and Humanitarian Aid (DGD/Belgium).

## Disclosure

This study was not preregistered in any independent institutional registry.

The analysis plan for this study was not preregistered in any independent institutional registry. All authors read and approved the final manuscript.

## Conflicts of Interest

The authors declare no conflicts of interest.

## Data Availability

Data can be made available upon reasonable request. Correspondence and requests for materials should be addressed to Branham Kitoko.
